# HES1 promotes extracellular matrix protein expression and inhibits proliferation and migration in human trabecular meshwork cells under oxidative stress

**DOI:** 10.18632/oncotarget.15631

**Published:** 2017-02-23

**Authors:** Linqi Xu, Yan Zhang, Ruru Guo, Wencui Shen, Yan Qi, Qingsong Wang, Zhenglong Guo, Chen Qi, Haifang Yin, Jiantao Wang

**Affiliations:** ^1^ Tianjin Medical University Eye Hospital, Tianjin Medical University Eye Institute, College of Optometry and Ophthalmology, Tianjin Medical University, Tianjin 300384, China; ^2^ Tianjin Research Center of Basic Medical Sciences and Department of Cell Biology, Tianjin Medical University, Tianjin 300070, China

**Keywords:** oxidative stress, glaucoma, trabecular meshwork, extracellular matrix, HES1

## Abstract

Glaucoma is the leading cause of irreversible blindness. The most prevalent form of glaucoma is primary open-angle glaucoma (POAG). Oxidative stress is one of the major pathogenic factors of the POAG, and can elicit molecular and functional changes in trabecular meshwork cells, causing increased aqueous humor outflow resistance and elevated intraocular pressure. However, the regulatory mechanisms underlying oxidative stress-induced cell phenotypic changes remain elusive. Herein, we exposed primary human trabecular meshwork cells to the oxidative stress induced by 300 μM H_2_O_2_ for 2 h, and found significantly up-regulated expression of extracellular matrix proteins and a transcription factor, hairy and enhancer of split-1 (HES1). The cell functions, including migration and proliferation, were impaired by the oxidative stress. Furthermore, HES1 shRNA abrogated the extracellular matrix protein up-regulation and rescued the functional defects caused by the oxidative stress; conversely, HES1 overexpression resulted in the molecular and functional changes similar to those induced by H_2_O_2_. These results suggest that HES1 promotes extracellular matrix protein expression and inhibits proliferative and migratory functions in the trabecular meshwork cells under oxidative stress, thereby providing a novel pathogenic mechanism underlying and a potential therapeutic target to the POAG.

## INTRODUCTION

Glaucoma is the leading cause of irreversible blindness, and afflicts more than 70 million people worldwide [1]. More than 90% of glaucoma patients suffer from primary open-angle glaucoma (POAG) [2]. In the POAG, elevated intraocular pressure (IOP) is regarded as a critical risk factor for disease progression [3, 4]. Although symptoms of the POAG can be relieved by lowering IOP [5, 6], the molecular mechanisms underlying IOP elevation remain elusive. Further insights into IOP regulatory mechanisms under pathological condition would facilitate development of a therapeutic modality to this vision-threatening disease.

Under normal condition, trabecular meshwork (TM) serves as the main regulator of IOP by controlling aqueous humor (AH) outflow [7, 8]. The extracellular matrix (ECM) proteins of TM cells undergo continuous turnover, and the equilibrium between production and degradation of the ECM proteins maintains a smooth drainage of AH [9, 10]. On the other hand, oxidative stress has been suggested as one of the major pathogenic factors causing increased resistance of AH outflow and elevated IOP in the POAG [9, 10], and the TM cell the most sensitive cell type to oxidant insults [12, 13]. Therefore, it would be plausible to speculate that oxidative stress could increase outflow resistance and elevate IOP during the POAG by promoting excessive production of the ECM proteins in the TM.

Then it would be interesting to ascertain the molecular link between oxidative stress and excessive ECM production in TM cells. Hairy and enhancer of split-1 (HES1) is a transcriptional repressor that belongs to the basic helix-loop-helix family of transcription factors [14]. HES1 functions downstream Notch signaling pathway, determining cell fate during development of nervous [14] and digestive systems [16]. More importantly, HES1 expression is up-regulated during lung [17, 18] and kidney [19] fibrosis, indicating an association of this transcription factor with profibrotic ECM protein production. Therefore, we hypothesize that HES1 may promote profibrotic ECM protein expression in TM cells under oxidative stress, contributing to the increased AH outflow resistance and elevated IOP that ultimately lead to the POAG. To test this hypothesis, we subjected human TM cells (HTMCs), a primary cell culture commonly used in glaucoma research [20–23], to oxidative stress induced by a sublethal dose of H_2_O_2_. We found the up-regulated expression of ECM and HES1 genes, as well as the compromised cell functions, such as migration and proliferation, in H_2_O_2_-stimulated HTMCs. Furthermore, our results demonstrated the requirement and sufficiency of HES1 up-regulation for the excessive ECM protein production and impaired cell functions in the HTMCs exposed to the oxidant, thereby providing a novel regulatory mechanism underlying the cell phenotypic changes caused by oxidative stress.

## RESULTS

### H_2_O_2_-induced oxidative stress impaired HTMCs’ viability

**Figure 1:**
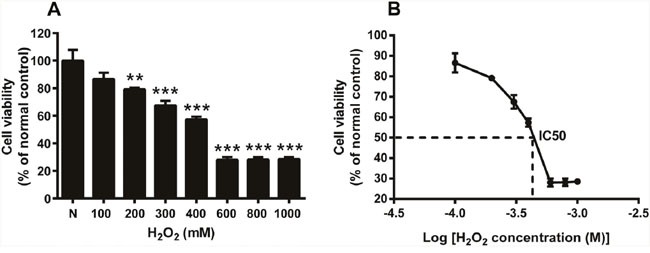
H_2_O_2_ at different concentrations induced oxidative stress and impaired the HTMC's viability The HTMCs were exposed to a series of concentrations of H_2_O_2_ for 2 h. The cell viability, expressed as percentage of normal control, was shown in **(A)**, N stands for normal control. A dose responsive curve was plotted in **(B)**, y-axis is the cell viability expressed as percentage of normal control, x-axis is logarithm of H_2_O_2_ concentration. The IC50 for H_2_O_2_ is 300 μM. The data were presented as mean ± SEM (n = 4 for each concentration in each experiment, and each experiment was repeated 3 times; ** *p* < 0.01, *** *p* < 0.001, as compared to normal control.)

To identify the effects of oxidative stress on cell viability, the HTMCs were subjected to different concentrations of H_2_O_2_ for 2 h. H_2_O_2_ at 100, 200, 300, and 400 μM reduced the cell viability progressively (Figure 1A, *p* < 0.01, 200 μM H_2_O_2_ vs normal; *p* < 0.001, 300 or 400 μM H_2_O_2_ vs normal), with the cell viability ranging from 86.55 ± 9.31% to 57.36 ± 3.97% of the normal control. This suggests that H_2_O_2_, at lower concentrations, inhibits the viability of HTMCs in a dose-dependent manner (Figure 1A). Moreover, the cell viability was reduced to 28.07 ± 3.95% of the normal control when the cells were exposed to 600 μM H_2_O_2_ (Figure 1A, *p* < 0.001, 600 μM H_2_O_2_ vs normal), however, it was not further compromised when H_2_O_2_ concentration increased to 800 and 1000 μM (Figure 1A), indicating a limited dose dependency of the H_2_O_2_-induced inhibition on cell viability. A dose-responsive curve was plotted to more clearly reflect the inhibitory effects of H_2_O_2_ on the cell viability (Figure 1B). The IC50 for H_2_O_2_ approximated 300 μM (Figure 1B). Since cell functions, including migration and proliferation, would be analyzed, the IC50 (H_2_O_2_ at 300 μM) was used in the following experiments.

### Oxidative stress up-regulated ECM protein expression and impaired cell functions

**Figure 2:**
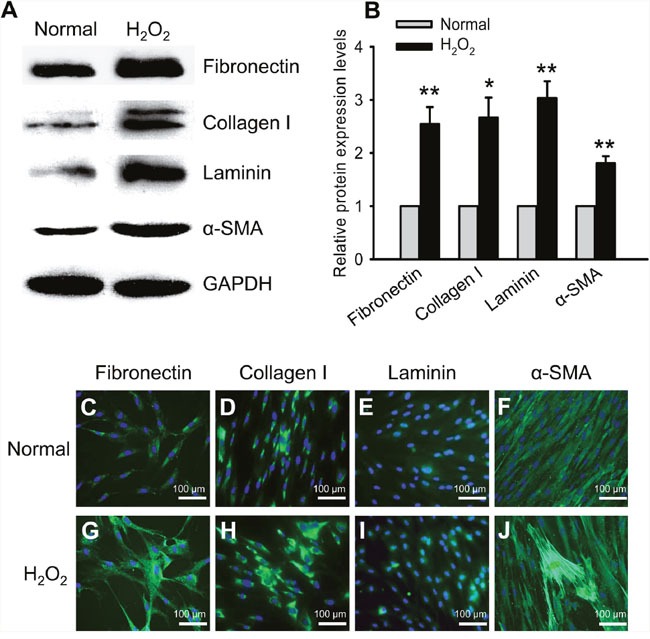
Oxidative stress promoted ECM protein expression Representativewestern blots showed up-regulated expression of the ECM proteins, including Fibronectin, Collagen I, Laminin, and α-SMA, in the HTMCs subjected to the 2 h-treatment of H_2_O_2_ **(A)**. The intensities of target protein bands were normalized to those of an internal standard, GAPDH, and the relative protein expression levels of the ECM genes were shown in **(B)**. Immunofluorescence confirmed the trends of up-regulated expression and showed cytoplasmic accumulation of the ECM proteins in the HTMCs under oxidative stress **(C-J)**. The data were presented as mean ± SEM (n = 3 per group for each experiment, each experiment was repeated 3 times; * *p* < 0.05, ** *p* < 0.01, as compared to normal control).

Treating the HTMCs with 300 μM H_2_O_2_ for 2 h significantly up-regulated the expression of profibrotic ECM proteins, including Fibronectin, Collagen I, Laminin, and α-SMA. As shown by western blots (Figure 2A), the relative protein levels of these ECM genes in the H_2_O_2_-treated cells were 1.81 ∼ 3.03 fold higher than the normal controls (Figure 2B, H_2_O_2_ vs normal, *p* < 0.01 for Fibronectin, Laminin, and α-SMA; *p* < 0.05 for Collagen I). These results were consistent with previous studies [23, 24]. The results of immunofluorescence revealed that the fluorescence intensities of the ECM proteins in the cytoplasm under oxidative stress were substantially greater than those under normal condition (Figure 2C-2J). Additionally, the HTMCs were more spread-out, and exhibited a patchy shape under H_2_O_2_ treatment (Figure 2G-2J), in contrast to a spindle-like shape observed in the normal control (Figure 2C-2F).

**Figure 3:**
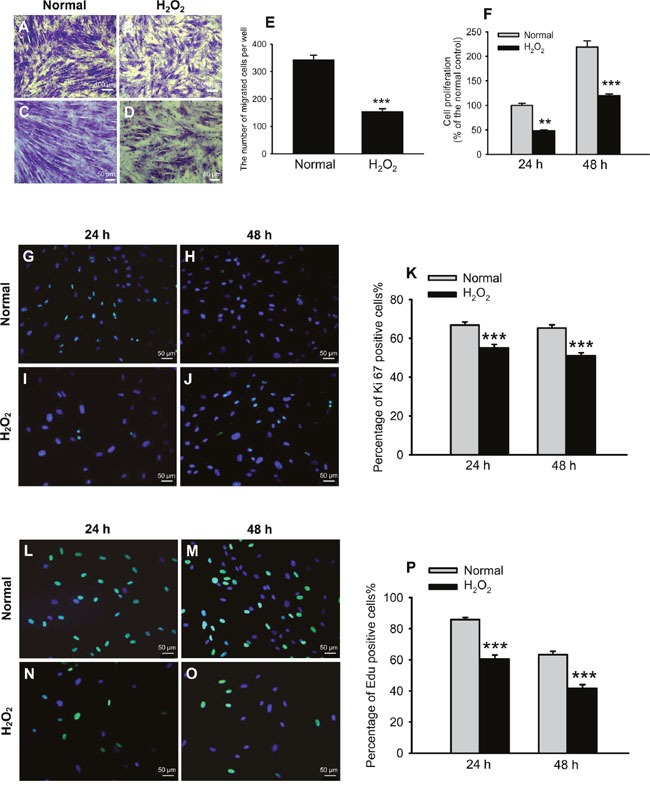
Oxidative stress impaired cell migration and proliferation Representative pictures of cell migration under norm and oxidative stress were shown in **(A-D)**, among which **(A)** and **(B)** are low magnification, **(C)** and **(D)** are high magnification. The migrated cells were quantified in **(E)**. Cell proliferation at 24 and 48 h after H_2_O_2_ exposure was first measured by CCK-8 and expressed as percentage of the normal control **(F)**. The effects of H_2_O_2_ on cell proliferation were confirmed by Ki67 staining and Edu assay. Representative pictures of Ki67 staining at 24 and 48 h post oxidative stress were shown in **(G-J)**. The percentages of Ki67-positive cells at both time points were quantified in **(K)**. Representative pictures of Edu assay were shown in **(L-O)**. The percentages of Edu-positive cells were quantified in **(P)**. The data were presented as mean ± SEM (n = 8 ∼ 32 per group for each experiment, each experiment was repeated 3 times; ** *p* < 0.01, *** *p* < 0.001, as compared to normal control).

We also evaluated cell migration and proliferation functions following 2 h-treatment of H_2_O_2_ (Figure 3). The cell migration was examined by a Transwell assay. The number of migrated cells was 343.00 ± 46.38 per well under normal condition, and reduced to 154.50 ± 27.68 pre well after H_2_O_2_ treatment, being only 45.04% of the normal control (Figure 3A-3E). On the other hand, the cell proliferation was first examined by a Cell Counting Kit-8 (CCK-8). The results showed that both the H_2_O_2_-treated HTMCs and the normal controls doubled their population from 24 to 48 h post the oxidative stress (Figure 3F). However, at each time point, the H_2_O_2_ exposure did significantly impair the cells’ proliferative ability, rendering the number of H_2_O_2_-treated cells only 48.48% and 54.60% of the normal control (Figure 3F, normal vs H_2_O_2_, *p* < 0.01 for 24 h; *p* < 0.001 for 48 h). To confirm the effects of H_2_O_2_ on cell proliferation, the HTMCs were then subjected to Ki67 staining and 5-ethynyl-2′-deoxyuridine (Edu) assay, which label a proliferation-associating nuclear protein [25] and actively synthesizing DNA [26], respectively. Indeed, the results demonstrated that both Ki67 and Edu signals were colocalized with 4′,6-diamidino-2-phenylindole (DAPI) staining (Figure 3G-3J and Figure 3L-3O), indicating their nuclear localizations. Importantly, in the H_2_O_2_-treated cells, the percentages of Ki67- and Edu-positive cells were both significantly lower than those in the normal controls at each time points (Figure 3K and 3P, normal vs H_2_O_2_, all *p* < 0.001). The results of Ki67 (Figure 3G-3K) and Edu staining (Figure 3L-3P), the 2 widely used approaches for detecting proliferative cells, were similar to those of CCK-8 (Figure 3F), therefore, CCK-8 was used in the following experiments to examine cell proliferation. Together, these results suggest that a transient oxidative exposure induces significant up-regulation and cytoplasmic accumulation of the profibrotic ECM proteins, and also generates relatively long-lasting inhibitory effects on migratory and proliferative functions in the HTMCs.

### Oxidative stress up-regulated HES1 expression at both transcript and protein levels

**Figure 4:**
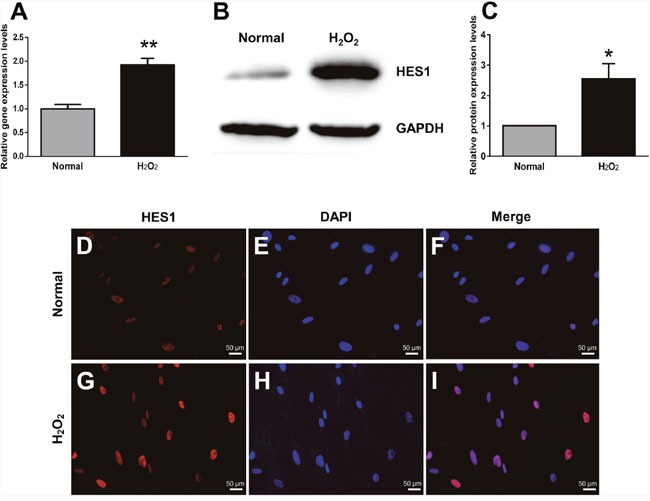
HES1 expression was up-regulated at both mRNA and protein levels in HTMCs under oxidative stress The qPCR showed an enhanced HES1 expression at mRNA levels in HTMCs subjected to the 2 h-treatment of H_2_O_2_ **(A)**. Representative western blots showed an increased protein expression of HES1 induced by the 2 h-oxidative stress **(B)**. The intensities of HES1 protein bands were normalized to those of the internal standard GAPDH, and the relative HES1 protein expression levels were shown in **(C)**. HES1 immunostaining confirmed the trend of its expression changes under norm **(D)** and oxidative stress **(G)**. The cell nuclei under both conditions were stained with DAPI (**E** and **H**). The HES1 staining was colocalized with DAPI staining (**F** and **I**). The data were presented as mean ± SEM (n = 3 per group for each experiment, each experiment was repeated 3 times; * *p* < 0.05, ** *p* < 0.01, as compared with normal control).

We then examined the expression of a transcription factor HES1 by quantitative real-time PCR (qPCR), western blot, and immunofluorescence in the HTMCs treated with H_2_O_2_. The qPCR results showed that HES1 mRNA levels increased 1.92 fold following oxidative stress (Figure 4A, *p* < 0.01, normal vs H_2_O_2_). Western blots showed that HES1 protein expression was boosted 2.55 fold by H_2_O_2_ treatment (Figure 4B and 4C, p < 0.05, normal vs H_2_O_2_). Immunofluorescence revealed a substantially greater fluorescence intensity of HES1 staining in the H_2_O_2_-treated cells than that in the normal controls (Figure 4D and 4G). Besides, HES1 staining colocalized with DAPI staining (Figure 4E and 4H), which suggests a predominant nuclear localization of this transcription under both normal and oxidative conditions (Figure 4F and 4I). Moreover, H_2_O_2_ induced a comparable extent of HES1 up-regulation at protein level to that at mRNA level, suggesting that the expression of this gene is mainly, if not entirely, regulated at the transcriptional level. Together with the results of elevated ECM protein levels following H_2_O_2_ treatment (Figure 2), the up-regulation of HES1 implicates its possible role in promoting ECM expression in the HTMCs under oxidative stress.

### HES1 knockdown abrogated the up-regulated ECM protein expression under oxidative stress

**Figure 5:**
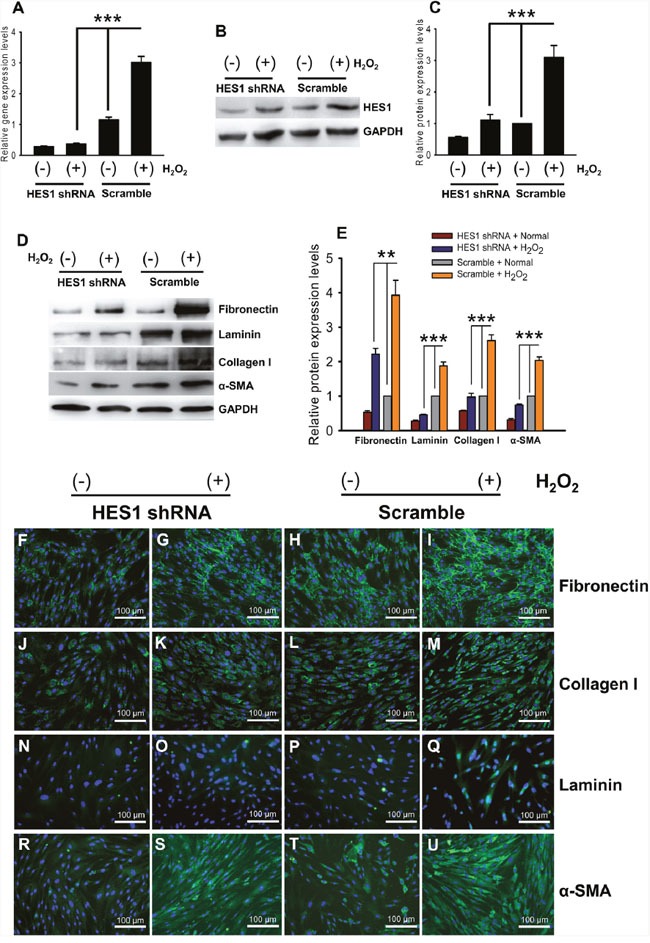
HES1 knockdown abrogated the up-regulation of ECM proteins in the HTMCs under oxidative stress HES1 shRNA efficiently knocked down HES1 expression at the transcript levels, as shown by qPCR, in the HTMCs under normal and oxidative stress conditions **(A)**. Western blots **(B)** and their quantification **(C)** showed the down-regulation of HES1 protein expression by HES1 shRNA in the HTMCs under both norm and oxidative stress. Western blots **(D)** revealed that the up-regulation of ECM proteins under oxidative stress was subdued (for Fibronectin), reversed (for Laminin), or abolished (for Collagen I and α-SMA) by HES1 shRNA **(E)**. Immunofluorescence confirmed the trends of ECM protein expression changes under both normal and oxidative conditions in the HES1 shRNA-transduced HTMCs **(F-U)**. The data were presented as mean ± SEM (n = 3 per group for each experiment, each experiment was repeated 3 times, ** *p* < 0.01, *** *p* < 0.001).

We then investigated whether HES1 knockdown could abrogate the H_2_O_2_-induced up-regulation of ECM protein expression in the HTMCs. Lentiviral particles carrying HES1 shRNA or a scrambled sequence were used to transduce the HTMCs. The cells were subjected to H_2_O_2_ treatment at 5 d after virus transduction. As expected, a significant up-regulation of HES1 gene expression after H_2_O_2_ exposure was detected by qPCR in the scramble-transduced cells (Figure 5A, *p* < 0.001, scramble normal vs scramble H_2_O_2_). More importantly, in the shRNA-transduced cells, HES1 mRNA levels were reduced to 24.50% of those in the scramble-transduced cells under normal condition (Figure 5A, *p* < 0.01, shRNA normal vs scramble normal); whereas the effects of shRNA were more dramatic under oxidative stress, with HES1 mRNA levels in the shRNA-transduced cells being merely 12.26% of those in the scramble-transduced cells (Figure 5A, *p* < 0.001, shRNA H_2_O_2_ vs scramble H_2_O_2_). HES1 protein expression exhibited a similar trend as shown by western blots (Figure 5B and 5C). These results confirmed an efficient knockdown of HES1 expression in the HTMCs at both basal and oxidative stress-induced levels.

Accordingly, under normal condition, there were 43% ∼ 73% reductions in the expression levels of ECM proteins in the HES1 shRNA-transduced cells, as compared to the scramble-transduced cells (Figure 5D and 5E), implicating that basal level expression of HES1 is required for maintaining normal ECM protein expression. Whereas under oxidative stress, the ECM proteins were up-regulated 3 ∼ 4 fold in the scramble-transduced cells as expected (Figure 5D and 5E, scramble normal vs scramble H_2_O_2_, *p* < 0.01 for Fibronectin; *p* < 0.001 for Laminin, Collagen I, and α-SMA). Yet, lentivirus-mediated expression of HES1 shRNA significantly subdued the H_2_O_2_-induced up-regulation of Fibronectin protein (Figure 5D and 5E, p < 0.01, shRNA H_2_O_2_ vs scramble H_2_O_2_), reversed that of Laminin protein (Figure 5D and 5E, p < 0.001, shRNA H_2_O_2_ vs scramble H_2_O_2_; *p* < 0.01, shRNA H_2_O_2_ vs scramble normal), and abrogated those of Collagen I and α-SMA proteins (Figure 5D and 5E, p < 0.001 for both ECM proteins, shRNA H_2_O_2_ vs scramble H_2_O_2_; *p* > 0.05 for both, shRNA H_2_O_2_ vs scramble normal). In addition, immunofluorescence staining confirmed the trends of protein expression changes detected by western blots, and revealed the cytoplasmic accumulation of these proteins (Figure 5F-5U). The results suggest that HES1 shRNA delivered by lentivirus can significantly reduce the expression of this transcription factor at both mRNA and protein levels, which inhibits or abolishes the up-regulation of ECM proteins in the HTMCs exposed to oxidative stress.

### HES1 knockdown partially rescued cell function defects under oxidative stress

**Figure 6:**
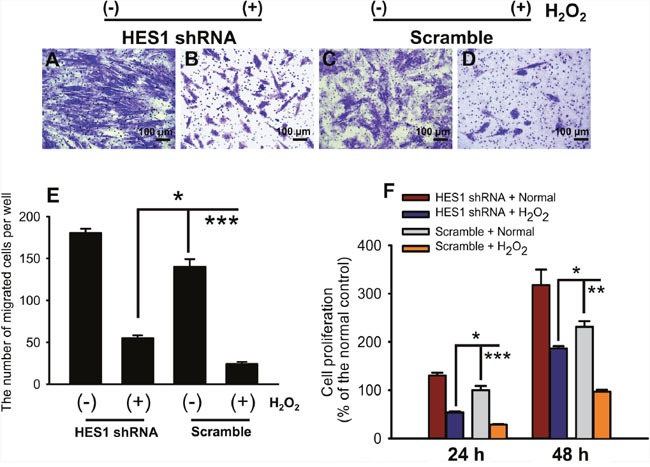
HES1 knockdown partially recovered cell functions under oxidative stress The representative pictures of cell migration were shown in **(A-D)** under both normal and oxidative stress conditions following HES1 shRNA transduction. HES1 shRNA partially recovered the impaired cell migration capacity caused by the 2 h-exposure to oxidative stress **(E)**. The diminished cell proliferation caused by the 2 h-treatment of H_2_O_2_ was partially (at 24 h following oxidative stress) or completely (at 48 h following oxidative stress) restored by HES1 shRNA transduction **(F)**. The data were presented as mean ± SEM (n = 3 ∼ 10 per group for each experiment, each experiment was repeated 3 times; * *p* < 0.05, ** *p* < 0.01, *** *p* < 0.001).

We next studied whether HES1 knockdown could ameliorate the cell function defects caused by oxidative stress. In the Transwell assay, the 2 h-treatment of H_2_O_2_ greatly reduced the number of migrated cells in the scramble-transduced HTMCs (Figure 6A-6E, *p* < 0.001, scramble normal vs scramble H_2_O_2_). Whereas knocking down HES1 expression by its shRNA significantly enhanced the cell's migratory ability under both normal and oxidative conditions (Figure 6A-6E, *p* < 0.001, shRNA normal vs scramble normal; *p* < 0.05, shRNA H_2_O_2_ vs scramble H_2_O_2_), however, the number of migrated cells in the shRNA-transduced HTMCs under oxidative stress was still significantly less than the normal control (Figure 6A-6E, *p* < 0.001, shRNA H_2_O_2_ vs scramble normal), indicating a partial rescue of cell migration by HES1 shRNA and involvement of additional regulators. Moreover, cell proliferation exhibited a similar trend. Knocking down HES1 expression significantly promoted cell proliferation at both 24 and 48 h after H_2_O_2_ treatment (Figure 5F, *p* < 0.05 for both time points, shRNA H_2_O_2_ vs scramble H_2_O_2_). It was notable that at 48 h following the oxidative episode, the number of shRNA-treated cells was comparable to the normal controls (Figure 6F, *p* > 0.05, shRNA H_2_O_2_ vs scramble normal), suggesting that down-regulating HES1 expression could restore the cell proliferative function during a prolonged period. The results of cell migration and proliferation assays indicate that HES1 up-regulation is required for the oxidative stress-induced cell function defects.

### HES1 overexpression generated the molecular and functional effects similar to oxidative stress

**Figure 7:**
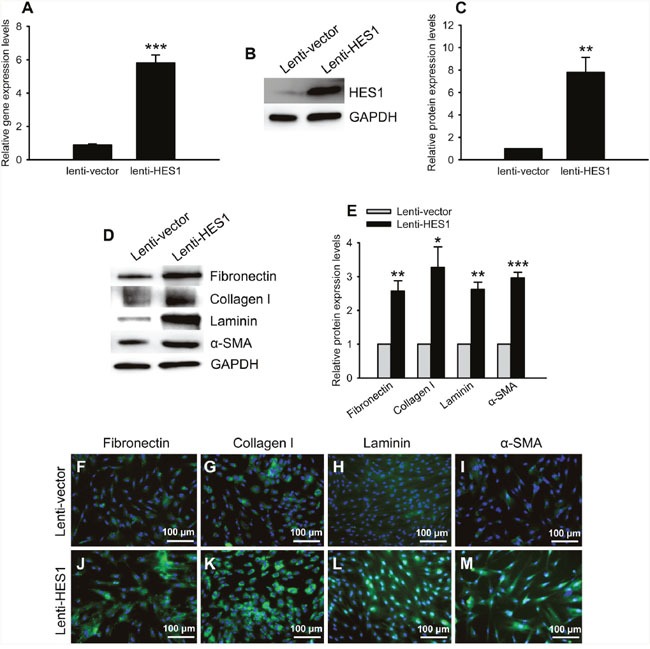
HES1 overexpression boosted expression of the ECM proteins The qPCR **(A)** and western blots **(B, C)** showed an augmented expression of HES1 at mRNA and protein levels, respectively, in the HTMCs transduced by the lentivirus carrying human HES1 cDNA. Representative western blots of the ECM proteins were shown in **(D)**. The western blot quantification showed the significant up-regulation of the ECM proteins **(E)**. Immunofluorescence confirmed the increased expression of the ECM proteins induced by HES1 overexpression **(F-M)**.

The HTMCs were transduced with a lentivirus carrying HES1 cDNA. At 5 d post virus transduction, the mRNA and protein levels of this transcription factor were up-regulated 6.55 and 7.81 fold, as shown by qPCR and western blots, respectively (Figure 7A-7C, lenti-HES1 vs lenti-vector, *p* < 0.001 for mRNA levels; *p* < 0.01 for protein levels), confirming an overexpression of HES1 gene in the HTMCs. The HES1 overexpression resulted in 2.57 ∼ 3.27 fold up-regulation of the profibrotic ECM proteins (Figure 7D and 7E, lenti-HES1 vs lenti-vector, *p* < 0.01 for Fibronectin and Laminin; *p* < 0.05 for Collagen I; *p* < 0.001 for α-SMA), and the up-regulated trends were corroborated by immunofluorescence (Figure 7F-7M).

**Figure 8:**
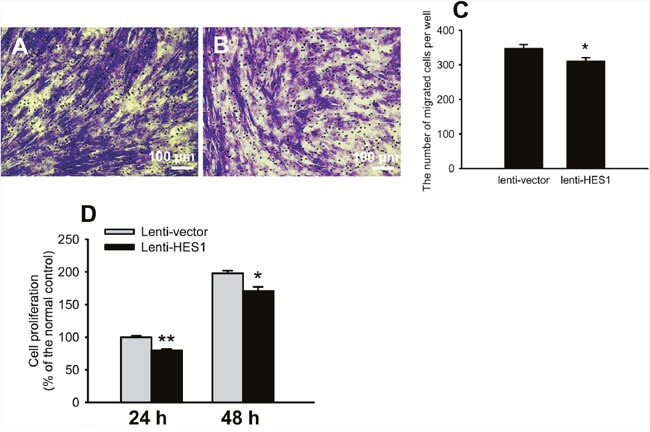
HES1 overexpression impaired the cell migratory and proliferative functions Representative pictures of cell migration were shown in **(A)** and **(B)**. Quantification showed that cell migration was reduced by overexpressing HES1 **(C)**. Cell proliferation was compromised at both 24 and 48 h following HES1 overexpression **(D)**. The data were presented as mean ± SEM (n = 3 ∼ 10 per group for each experiment, each experiment was repeated 3 times, * *p* < 0.05, ** *p* < 0.01).

Furthermore, the HTMCs overexpressing HES1 exhibited significantly diminished migration and proliferation, as compared to the empty vector-transduced cells (Figure 8, lenti-HES1 vs lenti-vector, *p* < 0.05 for Transwell; *p* < 0.01 for CCK at 24 h; *p* < 0.05 for CCK at 48 h). The effects of HES1 overexpression on ECM protein expression and cell functions resembled those produced by H_2_O_2_, suggesting that HES1 up-regulation is sufficient to elicit the cell phenotypic changes in ECM protein expression and cell functions as seen under oxidative stress.

## DISCUSSION

H_2_O_2_ is a key source of oxidant and natural constituent of AH in human eyes. The concentrations of H_2_O_2_ in AH of normal and cataractous eyes are 25 and 69 μM, respectively [27]. Nonetheless, it has been reported that patients with POAG have significantly elevated oxidative levels and reduced anti-oxidative capacity in AH as compared with cataract patients [28]. This implicates that TM cells in glaucomatous eyes might be exposed to H_2_O_2_ at higher concentration than that in cataract eyes, i.e. 69 μM. Whereas in the experimental studies, H_2_O_2_ at the concentrations ranging from 100 to 800 μM has been applied to the HTMCs to induce oxidative stress, serving as an *in vitro* pathological model for glaucoma research [23, 29, 30]. Therefore, in this study, H_2_O_2_ at the concentrations of 100, 200, 300, 400, 600, 800, and 1000 μM was transiently applied to the HTMC cultures, and cell viability was examined (Figure 1). An IC50 of H_2_O_2_ at 300 μM was selected to avoid massive cell death and loss of function caused by an overdose.

The up-regulation of the ECM proteins in the HTMCs upon an acute exposure to oxidative stress (Figure 2A and 2B) was consistent with the studies conducted by us [23] and others [24, 31]. The increased production and cytoplasmic accumulation of the ECM proteins (Figure 2C-2J) will likely result in an increased deposition of key ECM components, such as fibronectin, collagen I, and laminin, in TM. The excessive ECM deposition has been shown to remarkably alter TM microenvironment, resulting in an increased AH outflow resistance and elevated IOP as occurred in the POAG [9, 32, 33].

It has been reported that HES1 up-regulation under oxidative stress contributes to pathogenesis of multiple diseases, including endothelial cell injury in atherosclerosis and hypertension [34], neuronal cell apoptosis in Alzheimer's disease [35], and myocardial damage following ischemia and reperfusion [36]. Therefore, the enhanced HES1 expression in the HTMCs exposed to the transient oxidative stress (Figure 4) might unveil the pathogenic mechanism underlying the POAG. In addition, our results showed that the profibrotic ECM protein expression was up-regulated in the H_2_O_2_-treated HTMCs (Figure 2); knocking down HES1 expression by shRNA dramatically reduced the levels of these ECM proteins under both basal and oxidative conditions (Figure 5); and HES1 overexpression generated the opposite effects (Figure 7). These results, to our knowledge, are the first evidence showing HES1′s regulation on profibrotic ECM protein expression in the HTMCs under oxidative stress. The results also indicate HES1 as a possible therapeutic target to the POAG.

Furthermore, the transient exposure to oxidative stress impaired the important functions of HTMCs, including proliferation and migration (Figure 3). TM cells are indispensible components of AH outflow facility; whereas dwindling in TM cells causes an inadequate lining along outflow pathway and consequently the decreased AH outflow [13]. Additionally, each TM cell contains anti-oxidant defense machinery, TM's scavenging capacity deteriorates as its cell population shrinks [37], rendering TM cells more susceptible to oxidative insults. Both scenarios eventually lead to the elevated IOP and POAG [13]. By contrast, the relationship between HTMC migration and IOP elevation is less clear. However, it has been reported that a specific inhibitor of Rho-associated coiled coil-forming protein kinase, Y-27632, with a capacity of lowering IOP and increasing AH outflow in rabbits [38], promotes HTMC migration in a dose-dependent manner [39]. On the contrary, glucocorticoid, a drug known to reduce AH outflow, elevate the IOP, and induce secondary glaucoma, inhibits migration of HTMCs [40]. These reports suggest that there is at least an association, if not a causal relationship, between decreased cell migration and elevated IOP. Thus far, the diminished proliferative and migratory functions caused by the transient oxidative stress, together with the up-regulated ECM protein expression, may all contribute to IOP elevation and POAG incidence. Moreover, HES1 shRNA administration abrogated the ECM up-regulation and ameliorated the functional defects (Figure 5 and 6), thereby implicating a novel and effective therapeutic modality to the POAG that warrants further development.

Nonetheless, one of the limitations of this study is that the molecular mechanism remains not completely clear on how HES1 promotes the profibrotic ECM protein expression in the oxidative stress-treated HTMCs. One possibility could be that as a transcriptional repressor [14], HES1 may act on a negative regulator of the ECM gene expression, thereby de-repressing the ECM protein expression. Alternatively, as a signaling molecule in Notch pathway, HES1 might interact with other signaling molecules [41, 42], the resulting interaction or protein complex might activate the expression of ECM proteins in this particular type of cells under oxidative stress. Therefore, it would be interesting to employ RNA sequencing or mass spectrometry technology in future experiments to search for the downstream target or protein binding partner of HES1. Then it would also be important to prove HES1 target and the proposed molecular mechanism in an animal model of experimental glaucoma, such as the rat model induced by laser photocoagulation on TM.

In summary, the current study for first time showed that HES1 promotes the profibrotic ECM protein expression and inhibits proliferative and migratory functions in the HTMCs under oxidative stress. This study suggests a novel regulatory mechanism connecting oxidative stress with HTMC phenotypic changes at molecular and functional levels, and also hints on a therapeutic target to the POAG.

## MATERIALS AND METHODS

### Cell cultures

All experimental procedures were approved by the Laboratory Animal Care and Use Committee of Tianjin Medical University (Permit Number: SYXK 2009-0001) and in accordance with the Association for Research in Vision and Ophthalmology Statement for the Use of Animals in Ophthalmic and Vision Research. The HTMCs, the primary cell culture isolated from the juxtacanalicular and corneoscleral regions of human eye, were purchased from Sciencell Research Laboratories (San Diego, CA, USA). The detailed information of the HTMCs can be seen athttps://www.sciencellonline.com/human-trabecular-meshwork-cells.html#product_tabs_review_tabbed. The cells were maintained in the complete culture media composed of Dulbecco's Modified Eagle Medium (DMEM, 4.5 g/L glucose, Life Technologies, Grand Island, NY, USA), 10% Fetal Bovine Serum (FBS, Life Technologies, Grand Island, NY, USA), 100 U/ml penicillin/100 μg/ml streptomycin (Life Technologies, Grand Island, NY, USA), and 2 mM L-glutamine (Life Technologies, Grand Island, NY, USA) at 37 °C and 5% CO_2_ in a cell culture incubator (Thermo Scientific, Waltham, MA, USA).

### Oxidative stress

The HTMCs were seeded in a 96-well plate (8×10^3^ cells / well) and cultured in plain DMEM (4.5 g/L glucose, Life Technologies, Grand Island, NY, USA) for 24 h at 37 °C. The cells were treated with DMEM containing 100, 200, 300, 400, 600, 800, and 1000 μM H_2_O_2_ for 2 h. The cells treated with plain DMEM were included as the normal control. Then the media were replaced with 100 μl DMEM containing 10 μl CCK-8 reagent (Dojindo Laboratories, Kumamoto, Japan). The cells were incubated with CCK-8 reagent at 37 °C for 2 h. The absorbance at 450 nm (A450) was measured by a Tecan Infinite M200 multimode microplate reader (Tecan Group Ltd., Männedorf, Switzerland). The cell viability was expressed as percentage of normal control.

### RNA isolation and quantitative real-time PCR

Total RNA of the HTMCs was extracted by Trizol reagent (Life Technologies, Grand Island, NY, USA). The concentration and purity of total RNA were examined by a Nanodrop 2000 (Thermo Scientific, Waltham, MA, USA). After thorough digestion with DNase I (Thermo Scientific, Waltham, MA, USA), 1 μg of total RNA was reverse transcribed using random hexamer primers in a Transcriptor First Strand cDNA Synthesis Kit (Roche, Basel, Switzerland) according to the manufacturer's protocol.

The qPCR was performed in triplicate in a 10 μl mixture containing cDNA template, gene-specific primers (Table 1), and iTaq Universal SYBR Green Supermix (Roche, Basel, Switzerland) in a 7500 Fast Real-time PCR System (Applied Biosystems, Waltham, MA, USA). The cDNA content of each target gene was normalized to internal standard GAPDH gene. The serially diluted pooled cDNA samples were used as templates to generate a standard curve between Ct values of each gene and logarithm of cDNA template concentrations. The standard curves served as positive controls for qPCR, and suggested similar priming efficiency between each target gene and GAPDH gene. The reactions using water as template served as negative controls for qPCR. The PCR program consisted of an initial cycle of 95 °C for 10 min followed by 40 cycles of 95 °C for 30 s and 60 °C for 1 min. A dissociation stage was added to examine amplicon specificity. The relative expression levels of target genes were analyzed using a comparative threshold cycle (2^−ΔΔCt^) method.

**Table 1:** List of oligonucleotides in this study

Genes	Purposes	Sequences
GAPDH-F	qPCR primer	5′-CGAGATCCCTCCAAAATCAA-3′
GAPDH-R	qPCR primer	5′-GTCTTCTGGGTGGCAGTGAT-3′
HES1-F	qPCR primer	5′-TCAACACGACACCGGATAAA-3′
HES1-R	qPCR primer	5′-CCGCGAGCTATCTTTCTTCA-3′
Pac-F	qPCR primer	5′-CGAGTACAAGCCCACGGT-3′
Pac-R	qPCR primer	5′-GTTCTTGCAGCTCGGTGAC-3′
RPPH1-F	qPCR primer	5′-GCGGATGCCTCCTTTGC-3′
RPPH1-R	qPCR primer	5′-ACCTCACCTCAGCCATTGAACT-3′
HES1-F	Expression primer	5′-GCTCTAGAGCAATGCCAGCTGATATAATG-3′
HES1-R	Expression primer	5′-GGAATTCCCCTCAGTTCCGCCACGGCCT-3′
HES1	shRNA	5′-GAGCACAGAAAGTCATCAAAG-3′

### Plasmid construction

The shRNA against human HES1 gene (Table 1) was designed using web-based software at http://rnaidesigner.thermofisher.com/rnaiexpress. A scramble sequence (Addgene, Cambridge, MA, USA) was selected as a negative control. Both oligonucleotides were synthesized by Beijing Genomics Institute (Beijing, China), cloned into pLKO.1-puro shRNA vector (Addgene, Cambridge, MA, USA) via restriction endonuclease sites Age1 and EcoR1. The recombinant vectors were termed as pLKO.1-HES1 shRNA and pLKO.1-scramble.

The cDNA of HES1 gene was amplified by PCR using a commercially available plasmid (Addgene, Cambridge, MA, USA) as template. The PCR product was cloned into the pCDH-CMV-MCS-EF1α-puro lentiviral vector (Addgene, Cambridge, MA, USA) through Xho1 and EcoR1 sites. The resulting vector was designated as lenti-HES1. The empty vector was termed as lenti-vector and subserved a negative control.

### Lentivirus packaging, tittering, and transduction of HTMCs

The lentivirus was packaged and tittered as previously described [43] with modifications. Briefly, 293FT cells were seeded into 6-well plates at a density of 6×10^5^ cells / well, and cultured for 24 h before transfection. The 293FT cells were co-transfected with 2 μg lentiviral expression vector lenti-HES1, or lenti-vector, or pLKO.1-HES1 shRNA, or pLKO.1-scramble, 1.5 μg pPAX2 packaging plasmid, and 0.5 μg pVSVG envelope plasmid with assistance of 10 μl lipofectamine 2000 transfection reagent (Life Technologies, Grand Island, NY, USA). The culture media were replaced at 6 h post transfection. At 48 h after transfection, the culture media were collected and centrifuged at 1,500 rpm at room temperature for 10 min. The viral particle-containing supernatants (unconcentrated virus) were aliquoted and stored at -80 °C.

To measure the titers of packaged viruses, 293T cells seeded in a 6-well plate with a density of 8×10^5^ cells / well were transduced with 1, 10, and 100 μl of each unconcentrated virus with assistance of polybrene. At 64 h post transduction, DNase I (Thermo Scientific, Waltham, MA, USA) was applied to culture media to remove the possible residual plasmids from virus packaging. The cells were rinsed with pre-warmed phosphate buffer saline (PBS) twice. The genomic DNA of transduced cells was extracted by a GeneJET Genomic DNA Purification Kit (Thermo Scientific, Waltham, MA, USA), after which RNase (Thermo Scientific, Waltham, MA, USA) was used to eliminate RNA contamination. The concentration and purity of genomic DNA were examined by a Nanodrop 2000 (Thermo Scientific, Waltham, MA, USA). Then 100 ng genomic DNA was used as template of qPCR. The copy number of puromycin resistance gene (puromycin-N-acetyltransferase gene, pac) on the lentiviral vector represents the copy number of the viruses integrated into genome; whereas Ribonuclease P RNA component H1 gene (RPPH1) served as an endogenous reference that has 2 copies in each diploid cell of human origin. The primers of both genes were listed in Table 1. The copy number of pac gene was normalized to that of RPPH1 gene. The qPCR was performed as described above. The titer was calculated as the averaged number of cells that can be transduced by 1 ml virus. The viral titers were pLKO.1-HES1 shRNA, 6.58×10^7^ integration unit (IU)/ml; pLKO.1-scramble, 1.17×10^8^ IU/ml; Lenti-HES1, 9.15×10^7^ IU/ml; Lenti-vector, 1.26×10^8^ IU/ml.

The HTMCs were seeded in 24-well plates at a density of 2×10^5^ cells / well. The cells were transduced by the viruses with equally-adjusted titers in presence of polybrene. The media were replaced with fresh complete culture media at 20 h post transduction. The subsequent analyses, including gene expression and cell function assays, were conducted at 5 d following viral transduction.

### Western blots

Following the 2 h-H_2_O_2_ treatment, the HTMCs were washed with pre-chilled PBS and harvested on ice. Total protein was extracted using a lysis buffer (pH 6.8, CWBIO, Beijing, China) supplemented with 5% β-mercaptoethanol (Sigma-Aldrich, St. Louis, MO, USA). Protein concentration was determined using a Bicinchoninic Acid Protein Assay Kit (CWBIO, Beijing, China). Western blots were preformed as previously described [44]. Briefly, 50 μg protein samples were denatured at 100 °C for 10 min, separated on an 8% or 12% sodium dodecyl sulfate polyacrylamide gel, and transferred to a polyvinylidene difluoride (PVDF) membrane. The PVDF membranes were washed, blocked with 5% non-fat milk for 1 h at room temperature, and then incubated overnight at 4 °C with primary antibodies, including rabbit polyclonal anti-HES1(1:250, Abcam, Cambridge, MA, USA), mouse monoclonal anti-Fibronectin (1:5000, Abcam, Cambridge, MA, USA), mouse monoclonal anti-α-SMA (1:1000, Sigma-Aldrich, St. Louis, MO, USA), rabbit polyclonal anti-Collagen I (1:2000, Abcam, Cambridge, MA, USA), and mouse monoclonal anti-Laminin-5 (1:500, R&D Systems, Minneapolis, MN, USA). On the next day, the membranes were washed and incubated with corresponding horseradish peroxidase-conjugated secondary antibodies (Tianjin Sungene Biotech Co., Ltd., Tianjin, China) at room temperature for 2 h. Following intensive washes, protein signals were visualized by enhanced chemiluminescence plus reagents (Millipore, Billerica, MA, USA). The images were captured using a Multispectral Imaging System (Biospectrum AC Chemi HR 410, UVP, LLC, Upland, CA, USA). The blots were then stripped and incubated with a mouse monoclonal antibody to GAPDH (1:5000, Cell Signaling Technology, Danvers, MA, USA) to subserve an internal standard. The optical densities of target proteins were quantified by Quantity One (Bio-Rad, Hercules, CA, USA) and normalized to those of GAPDH.

### Immunofluorescence

The HTMCs were seeded on coverslips in a 24-well plate with a density of 8×10^4^ cells / well. The cells were subjected to oxidative stress for 2 h, and washed with pre-warmed Dulbecco's Phosphate Buffered Saline (DPBS) twice, fixed with 4% paraformaldehyde (PFA) for 30 min at 4 °C. The cells were permeabilized with 0.1% Triton X-100 (Sigma-Aldrich, St. Louis, MO, USA) in DPBS for 30 min and blocked with 5% goat serum (Life Technologies, Grand Island, NY, USA) for 2 h at room temperature. The cells were then incubated overnight at 4 °C with primary antibodies, including anti-Fibronectin (Abcam, Cambridge, MA, USA), anti-Collagen I (Abcam, Cambridge, MA, USA), anti-Laminin-5 (R&D Systems, Minneapolis, MN, USA), anti-α-SMA (Sigma-Aldrich, St. Louis, MO, USA), and anti-HES1 (Abcam, Cambridge, MA, USA)(all diluted at 1:100). The HTMCs were washed and incubated with Alexa 488-conjugated secondary antibodies (Life Technologies, Grand Island, NY, USA) for 2 h at room temperature. Afterwards, the coverslips were mounted with ProLong Gold Antifade with DAPI (Life Technologies, Grand Island, NY, USA). The pictures were taken by the cellSens Standard electronic system (Olympus Optical Co. Ltd., Tokyo, Japan) under a fluorescence microscope (BX51, Olympus Optical Co. Ltd., Tokyo, Japan) using identical optical parameters.

### Migration assay

The HTMCs, either cultured under normal condition or at 5 d post viral transduction, were treated with H_2_O_2_ for 2 h. Then the cells were digested and counted with Trypan Blue staining. The live cells were plated in 6-well plates at a density of 1×10^6^ cells / well, and incubated in serum free DMEM (4.5 g/L glucose, Life Technologies, Grand Island, NY, USA) at 37 °C for 24 h. Afterwards, migration assay was performed using Transwell chambers in a 24-well plate format (Corning Costar, Cambridge, MA, USA). The chambers were balanced with DMEM (4.5 g/L glucose, Life Technologies, Grand Island, NY, USA) for 1 h at 37 °C. Each upper chamber was seeded with 1.5×10^5^ HTMCs in 100 μl DMEM (4.5 g/L glucose, Life Technologies, Grand Island, NY, USA), and the lower chamber was filled with 500 μl complete culture media. At 10 h after seeding, the cells were briefly rinsed with PBS, fixed and stained with 0.1% crystal violet (dissolved in anhydrous ethanol, Sigma-Aldrich, St. Louis, MO, USA) for 20 min, and washed 3 times with water. The cells at inner bottom of upper chamber were wiped off with a damped cotton swab. The membranes of upper chambers were air dried, circumcised, and mounted on the slides with neutral balsam (Sigma-Aldrich, St. Louis, MO, USA). The pictures were taken using the cellSens Standard electronic system (Olympus Optical Co. Ltd., Tokyo, Japan) under a light microscope (BX51, Olympus Optical Co. Ltd., Tokyo, Japan). The membrane was divided into 4 quadrants, and 3 representative pictures were acquired for each quadrant. The numbers of cells on the 3 pictures were averaged to represent cell migration in each quadrant, and the averages representing 4 quadrants were added up to represent cell migration on each membrane.

### Proliferation assays

The HTMCs, either cultured under normal condition or at 5 d post viral transduction, were subjected to the oxidative stress followed by Trypan Blue counting as described in Migration assay.

#### CCK-8 assay

The cells were seeded at a density of 5×10^3^ cells / well in complete culture media in a 96-well plate. At 24 and 48 h after seeding, the complete culture media were replaced with 100 μl DMEM (4.5 g/L glucose, Life Technologies, Grand Island, NY, USA) containing 10 μl CCK-8 reagent (Dojindo Laboratories, Kumamoto, Japan). The A450 was measured as described above. Cell proliferation was expressed as percentage of the normal control.

#### Ki67 staining

The cells were seeded at a density of 2×10^4^ cells / well on coverslips in a 24-well plate. The cells were cultured in complete culture media for 24 or 48 h. Then the cells were fixed with 4% PFA, washed with DPBS, and treated with the blocking and permealization buffer (0.1% Tween 20, 1% bovine serum albumin (BSA), 10% rabbit serum, and 0.3M glycine in DPBS, Sigma-Aldrich, St. Louis, MO, USA) at room temperature for 1 h. The cells were incubated with the primary antibody rabbit polyclonal anti-Ki67 (diluted at 1:1000, Abcam, Cambridge, MA, USA) at 4 °C overnight. The cells were incubated with Alexa 488-conjugated secondary antibody at room temperature for 1.5 h. Then the coverslip mounting and picture taking were performed as described in Immunofluorescence. The quantification of Ki67-positive staining was conducted as described in Migration assay.

#### Edu assay

The cells were seeded on coverslips in a 24-well plate in complete culture media at a density of 2×10^4^ cells / well. Then Edu assay was performed using a Click-iT Edu imaging Kit (Thermo Scientific, Waltham, MA, USA). When the cells were cultured at 24 or 48 h, 10 μM Edu was added to label the synthesizing DNA. Twenty hours after addition, the integrated Edu was detected according to the manufacturer's protocol. The coverslip mounting, picture taking, and quantification of Edu-positive staining was performed as described above.

### Statistical analyses

The data were analyzed by Graphpad (GraphPad Software, Inc., La Jolla, CA, USA) software. The data were examined by D'Agostino and Pearson omnibus normality test, those with Gaussian distribution were examined by Levene test to confirm homogeneity of variance, and then analyzed by unpaired *t* test or One-way or Two-way ANOVA followed by *Tukey post hoc*; the data with nonparametric distribution were analyzed by Kruskal–Wallis test followed by *Dunn's post hoc*. A *p* value less than 0.05 was considered significant.
